# Draft Genome Sequence of the Yeast Nadsonia starkeyi-henricii UCD142, Isolated from Forest Soil in Ireland

**DOI:** 10.1128/genomeA.00549-18

**Published:** 2018-06-21

**Authors:** Sinéad O’Boyle, Sean A. Bergin, Éabha E. Hussey, Aaron D. McLaughlin, Luan R. Riddell, Kevin P. Byrne, Kenneth H. Wolfe, Caoimhe E. O’Brien, Geraldine Butler

**Affiliations:** a School of Biomedical and Biomolecular Sciences, Conway Institute, University College Dublin, Dublin, Ireland; b School of Medicine, Conway Institute, University College Dublin, Dublin, Ireland

## Abstract

We report a draft genome sequence of a strain of the nonfermentative yeast Nadsonia starkeyi-henricii, isolated from soil in a forest in Ireland. Comparison to Nadsonia fulvescens shows few rearrangements and a level of divergence similar to that of Saccharomyces cerevisiae versus Saccharomyces paradoxus. Its mitochondrial genome lacks *NAD* genes.

## GENOME ANNOUNCEMENT

Nadsonia starkeyi-henricii (1) is a little-studied yeast. It is one of the four accepted species and varieties in the genus Nadsonia and was formerly called Schizoblastosporion starkeyi-henricii (2–4). It is unable to ferment sugars and is only able to grow by respiration (4).

Nadsonia species have lower maximum temperatures for growth than most other yeasts, which may have caused them to be overlooked in previous surveys of yeast diversity. Most previous isolates of N. starkeyi-henricii were found in soils from forests or bogs, mainly in Northern Europe (2, 3, 5). It will not grow at temperatures above 25 to 30°C, and it dies at 35°C (6). Nadsonia species are characterized by their unusual mode of mitotic cell division, with buds forming alternately from opposite poles of the cell. Sporulation has never been observed in N. starkeyi-henricii, unlike the other members of this genus. Phylogenetically, Nadsonia is in the Yarrowia clade, distantly related to Saccharomyces cerevisiae (7, 8). One genome sequence has been published from this genus, Nadsonia fulvescens var. elongata (7).

Strain UCD142 was isolated from soil in a coniferous forest in Dublin, Ireland (global positioning satellite [GPS] coordinates N53.241645, W6.294017). It was cultured at room temperature on yeast extract-peptone-dextrose (YPD) agar plates containing chloramphenicol (3% wt/vol) and carbenicillin (10% wt/vol). Genome sequencing (6.6 million paired-end reads of 150 bp) was performed by BGI Tech Solutions (China) with an Illumina HiSeq 4000. The genome was assembled into 318 contigs (60× coverage, >1 kb) using SPAdes v 3.11.1 (9).

The N. starkeyi-henricii genome assembly is 14.6 Mb, similar to Nadsonia fulvescens (13.7 Mb; reference 7). Half the data are present in the 40 scaffolds (*L*_50_) that are larger than 120 kb (*N*_50_), and the largest scaffold is 404 kb. BWA and SAMtools (10, 11) identified only 2,337 single nucleotide polymorphisms (SNPs), and the genome appears to be haploid. Annotation using YGAP (12) predicted 5,216 protein-coding genes, which compares to 5,657 in N. fulvescens (7). tRNAscan-SE (13) predicted 607 tRNA genes in the nuclear genome, an unusually high number for a yeast. Similar to N. fulvescens, the 5S rRNA genes are not located within the repeating 18S-5.8S-26S array but instead are dispersed at approximately 115 locations (7).

Comparison of the 10 largest N. starkeyi-henricii scaffolds to N. fulvescens by BLAST and dot-matrix plots showed that the genomes are mostly collinear. Protein sequence divergence between the two Nadsonia species is approximately equal to that between S. cerevisiae and S. paradoxus (e.g., 89% amino acid sequence identity in *MDN1*, the largest gene in the genome).

The mitochondrial genome is a 22,069-bp circle containing 8 protein-coding genes with no introns. It does not contain any *NAD* genes for NADH dehydrogenase subunits, in contrast to Yarrowia lipolytica (14), indicating an absence of mitochondrial respiratory complex I. Complex I is present in most yeasts but has been lost in three clades centered on Saccharomyces, Schizosaccharomyces, and Nadsonia (7).

### Accession number(s).

This whole-genome shotgun project has been deposited in DDBJ/ENA/GenBank under accession no. QBLK00000000. The version described in this paper is the first version, QBLK01000000.
